# Osteochondral Autograft Transplantation Coupled With Platelet-Rich Plasma and Hyaluronic Acid Injections Can Yield Favorable Outcomes in Patients With Osteochondral Lesions of the Talus

**DOI:** 10.1016/j.asmr.2025.101206

**Published:** 2025-06-26

**Authors:** George A. Rublev, Nivedita Pant, Mohamed Ahmed Mohamed, Tamaz Tamazishvili, Giorgi Khokhashvili, Irakli Gogua, Irakli Kartozia, Giorgi Zimlitski, George Loria, Vazha Gaprindashvili, Mikheil Zimlitski, Levan Natchkebia

**Affiliations:** a From Center for Arthrology, Sports Medicine, and Regenerative PRP at MediClub, Tbilisi, Georgia; b AIETI David Tvildiani Medical University, Tbilisi, Georgia

## Abstract

**Purpose:**

To evaluate the outcomes of patients who had osteochondral lesions of the talus treated with osteochondral allograft transplants combined with platelet-rich plasma (PRP) and hyaluronic acid (HA).

**Methods:**

We aimed to investigate the outcomes of patients who underwent osteochondral autograft transplantation surgery (OATS) from 2015 to 2022. The inclusion criteria for patients to undergo OATS included a defect size greater than 5 mm and failure of conservative management. The preoperative and postoperative visual analog scale (VAS) and American Orthopaedic Foot & Ankle Society (AOFAS) scores were recorded and subsequently analyzed using a paired *t* test. We also performed cohort-specific analyses that included the minimal clinically important difference, patient acceptable symptom state, and substantial clinical benefit. PRP injections were administered at 8 weeks postoperatively and were given every 2 weeks, for 3 total injections. HA was injected once every 6 months for the following 2 years. The minimum follow-up time was 2 years.

**Results:**

At the 12-month follow-up, all 19 patients reported a mean increase in the VAS score of 33.40 (95% confidence interval, 30.9-35.8). The average age was 31 years, and there were 12 male and 7 female patients. Of the 19 patients, 17 reported no restriction of motion, whereas the other 2 patients reported some restriction of dorsiflexion. Ankle function based on the AOFAS scoring system showed good to excellent results in 18 of 19 cases (94%), with no long-term donor-site morbidity and a mean increase of 37.49 (95% confidence interval, 34.7-40). Hardware removal of lag screws was conducted at 12 months after initial surgery; all cases resulted in union of the malleolar osteotomy. The average follow-up time was 3 years.

**Conclusions:**

Combining OATS with PRP-HA injections can yield promising results for patients with osteochondral lesions of the talus, showing significant improvement in VAS and AOFAS scores postoperatively.

**Level of Evidence:**

Level IV, therapeutic case series.

Osteochondral lesions of the talus (OCLTs) represent a challenging orthopaedic condition with potentially significant morbidity.^1^ They are characterized by disruption of the articular cartilage and underlying subchondral bone in the ankle joint.^2^ The location of OCLTs is mapped by the Raikin grid classification, which divides the talar dome into 9 zones.^3^^,^^4^ Studies suggest that defect size and diameter (as seen on magnetic resonance imaging [MRI]) are important determinants for conservative treatment failure; lesions larger than 10 mm in diameter are less likely to respond to treatments such as microfracture or drilling and may require more intensive procedures such as osteochondral autograft transplantation surgery (OATS).^5^, ^6^, ^7^

Regardless of defect size, first-line treatment of OCLTs consists of nonoperative conservative management, which includes nonsteroidal anti-inflammatory drugs, rest, immobilization, and physical therapy.^3^^,^^8^ For smaller, nondisplaced lesions, conservative management may lead to positive outcomes, not requiring surgery.^8^ Some evidence has supported the use of platelet-rich plasma (PRP) injections, along with nonoperative modification, as a form of adjunctive therapy; however, long-term studies of outcomes are lacking.^3^^,^^8^

Conservative treatment shows favorable clinical outcomes in patients with stage 1 or 2 OCLTs per the Berndt and Harty classification.^3^^,^^9^^,^^10^ Surgical treatment is considered after failure of conservative management strategies or for Berndt and Harty stage 2 to 5 lesions and is broadly based on repair, regeneration, and replacement of the cartilage segment.^11^, ^12^, ^13^, ^14^ Cartilage repair and regeneration techniques include microfracture and autologous chondrocyte implantation, which is often considered after failed microfracture.^15^ Microfracture is indicated for lesions measuring less than 1 to 1.5 cm, whereas autologous chondrocyte implantation can be used for larger lesions.^3^^,^^12^^,^^13^ Additional surgical treatment involving replacement of the defective cartilage with true hyaline cartilage, such as osteochondral autologous transplantation (e.g., OATS), is indicated for a defect size greater than 8 mm, failed reparative techniques, and stage 5 cystic lesions.^16^

Surgical intervention is commonly required for lesions that fail conservative management and for larger lesions (>8 mm) or lesions associated with persistent symptoms because these are unlikely to resolve on their own and would require operative treatment, especially in adults.^17^ Surgical treatment options may include arthroscopy with the removal of loose fragments, debridement, bone marrow stimulation using a microfracture technique, or other surgical interventions such as chondroplasty, OATS (including mosaicplasty), or osteochondral allograft transplantation from a non-weight-bearing or less weight-bearing portion of the knee to the ipsilateral talus.^1^^,^^3^^,^^17^, ^18^, ^19^

The use of PRP as an adjunct to microfracture treatment of OCLTs has shown good outcomes across multiple studies.^20^, ^21^, ^22^, ^23^ Preclinical studies have shown promising results combining PRP with OATS; however, the evidence in clinical practice remains sparse.^24^, ^25^, ^26^ The use of hyaluronic acid (HA) as an adjunct for the treatment of OCLTs when combined with microfracture has been described; however, no clinical studies have investigated the application of HA along with OATS or PRP plus HA along with OATS for the treatment of OCLTs.^27^

The purpose of this study was to evaluate the outcomes of patients who had OCLTs treated with osteochondral allograft transplants combined with PRP and HA. We hypothesized that OATS with PRP-HA injections would yield favorable outcomes when treating OCLTs at a minimum 2-year follow-up.

## Methods

The clinical, radiologic, and operative records of all patients treated for OCLTs by a single surgeon (L.N) from 2015 to 2022 at a single private center were reviewed retrospectively; this was not a consecutive series of all our patients with OCLTs. The diagnosis of OCLTs was based on clinical examination findings, along with preoperative radiographs and MRI scans of the ankle joint. Duration of symptoms and mechanism of injury, if any, were considered. Patient demographic characteristics, including age, sex, weight, and body mass index, were recorded (Table 1). The visual analog scale (VAS) score and American Orthopaedic Foot & Ankle Society (AOFAS) score were also recorded preoperatively and at the 12-month follow-up.

**Table 1:** Patient Sex, Age, BMI, Date of Surgery, and Radiologic Classification

Patient	Sex/Age, yr	Year of Procedure	BMI	Lesion Location (9-Grid Scheme)	Defect Size, mm	Procedure	No. of Grafts Used	Stage	
								Berndt and Harty Radiographic Staging System	Hepple MRI Staging System
Patient 1	M/33	2019	28.4	3, 6	<8	Chondroplasty + OATS	1	2	2b
Patient 2	M/53	2019	30.1	4	<8	Chondroplasty + OATS + osteotomy	1	2	3
Patient 3	M/31	2019	24.8	4, 7	8-16	Chondroplasty + OATS + osteotomy	2	3	4
Patient 4	M/33	2020	21.9	4	<8	OATS + osteotomy	1	2	2b
Patient 5	M/25	2019	24.8	4	<8	OATS + osteotomy	1	2	2b
Patient 6	M/25	2016	25.4	4, 7	8-16	Chondroplasty + OATS + osteotomy	2	3	3
Patient 7	M/39	2017	33.7	4	<8	Chondroplasty + OATS + osteotomy	1	2	2b
Patient 8	M/23	2017	23.1	4, 7	8-16	Chondroplasty + OATS + osteotomy	2	3	3
Patient 9	M/34	2020	32.1	4	<8	Chondroplasty + OATS + osteotomy	1	2	2b
Patient 10	M/28	2018	32	4, 7	8-16	Chondroplasty + OATS + osteotomy	2	3	2b
Patient 11	F/24	2020	23	4, 7	<8	Chondroplasty + OATS + osteotomy	1	2	2b
Patient 12	F/23	2020	26	4, 7	8-16	Chondroplasty + OATS + osteotomy	2	3	3
Patient 13	F/21	2020	24.8	7	<8	OATS + osteotomy	1	2	2b
Patient 14	F/23	2019	25	4	<8	OATS + osteotomy	1	2	2b
Patient 15	F/51	2019	34.1	4, 7	8-16	Chondroplasty + OATS + osteotomy	2	4	4
Patient 16	F/31	2020	24.6	4, 7	8-16	Chondroplasty + OATS + osteotomy	2	3	3
Patient 17	F/45	2021	21	4, 7	8-16	Chondroplasty + OATS + osteotomy	2	3	2b
Patient 18	M/20	2022	32	4	8-16	Chondroplasty + OATS + osteotomy	2	3	3
Patient 19	M/27	2022	26.9	4	8-16	Chondroplasty + OATS + osteotomy	2	3	2a

BMI, body mass index; F, female; M, male; MRI, magnetic resonance imaging; OATS, osteochondral autograft transplantation surgery.

**Fig 1:**
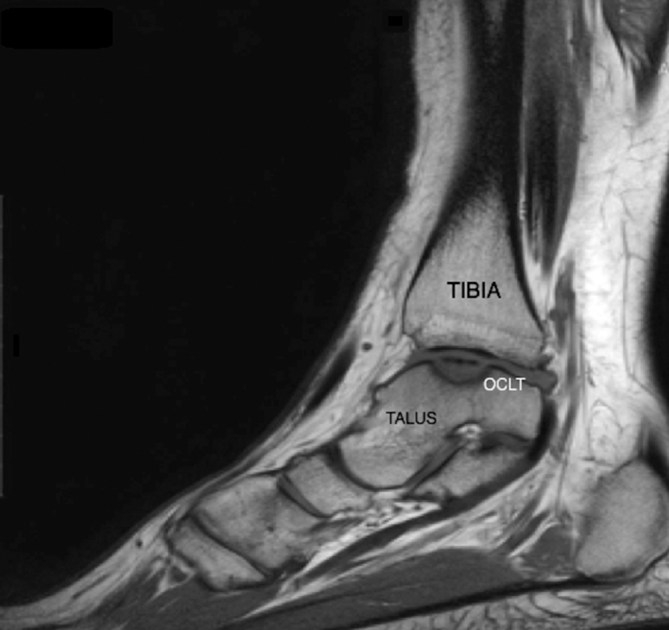
Preoperative sagittal magnetic resonance imaging scan showing a medial osteochondral lesion of the talus (OCLT). (1m, 1mm slices).

**Fig 2:**
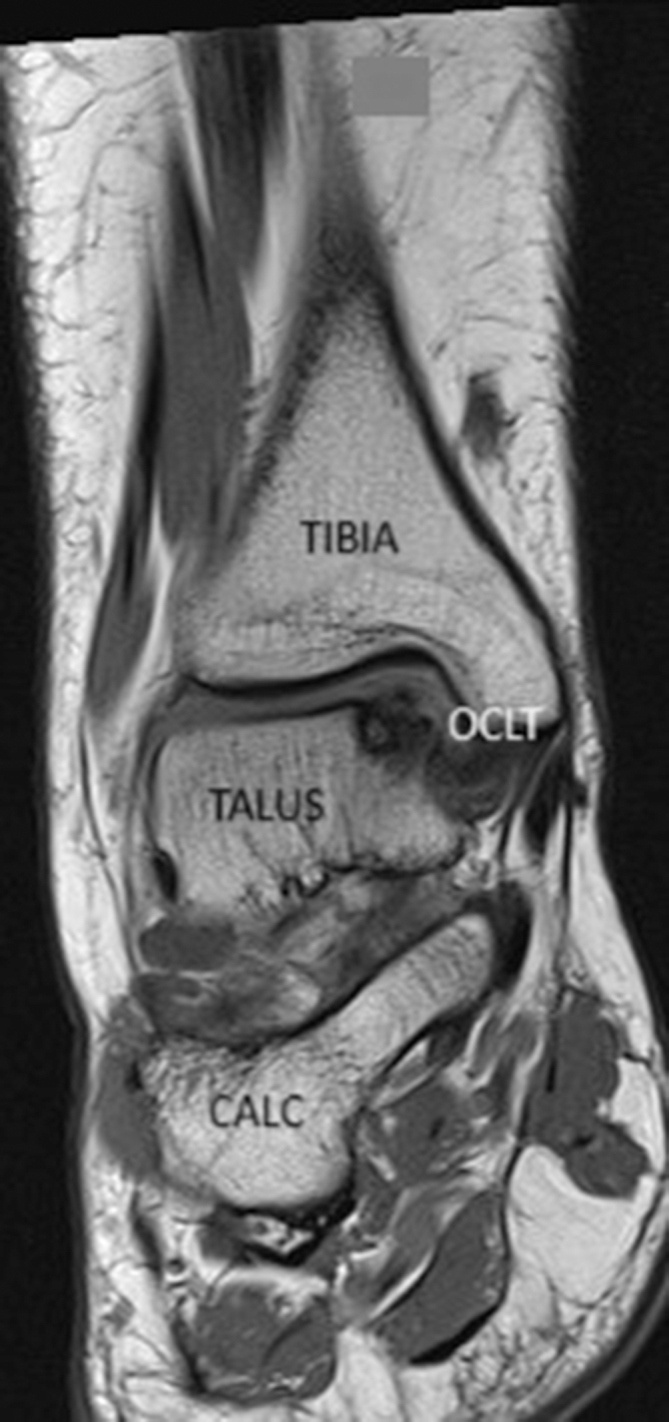
Preoperative anteroposterior magnetic resonance imaging scan showing a medial osteochondral lesion of the talus (OCLT). (CALC, Calcaneus)

**Fig 3:**
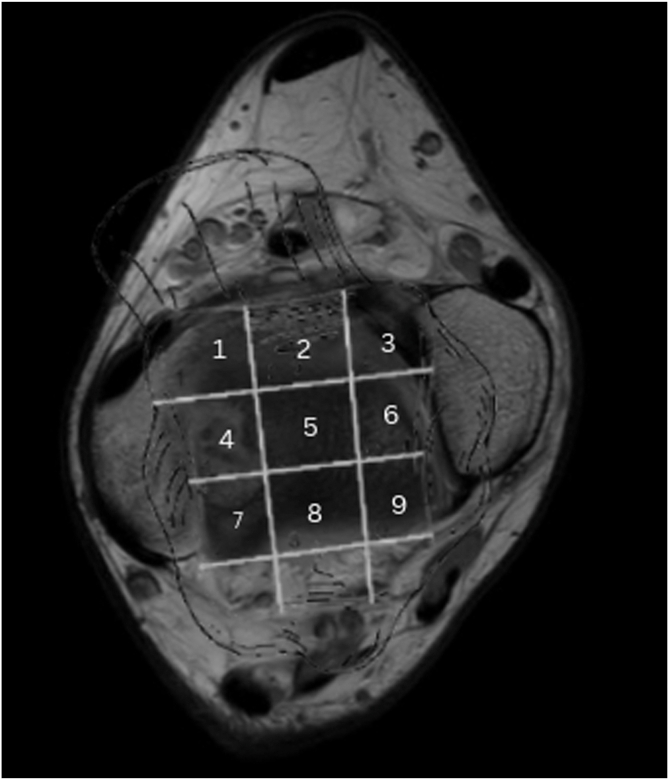
Raikin grid imposed on axial magnetic resonance imaging scan of talus. Lesions in locations 1, 4, and 7 are medial lesions; those in locations 3, 6, and 9 are medial lesions; and those in locations 2, 5, and 8 are central lesions.

Preoperatively, the clinical evaluation also involved assessing the donor site and examining radiographs of the lateral femoral condyle for any signs of degeneration that could increase the risk of donor-site morbidity, such as joint space narrowing or irregular joint contours.^11^^,^^28^ The talus and tibiotalar joint MRI scans were analyzed to assess the cartilage surface integrity and precisely locate the defect (Figs 1 and 2).^9^ The lesion was localized using the Raikin anatomic 9-zone grid system (Fig 3).^4^ Classification and staging of each patient’s preoperative imaging were completed using the Berndt and Harty radiographic system and the Hepple MRI system.^13^

The indications for patients to undergo OATS included a defect size greater than 5 mm. Patients with a defect size greater than 5 mm but less than 8 mm underwent OATS alone, whereas those with a defect size exceeding 8 mm received OATS with chondroplasty.^29^ We included patients who experienced acute trauma or who had chronic post-traumatic lesions, and none of the patients in our cohorts presented with OCLTs after an acute fracture. Additional inclusion criteria comprised the presence of ankle pain with a limited range of motion and failure of conservative management for a duration exceeding 6 months.^25^ We excluded patients younger than 18 years or older than 60 years and patients with a defect size less than 5 mm. We used the 5-mm cutoff because lesions less than 5 mm were considered too small for OATS and were instead treated with microfracture, which has been shown to achieve similar results for lesions of this size and is less invasive.^29^

Surgical outcomes were thoroughly evaluated by comparing patients’ preoperative and postoperative scores after undergoing OATS. To assess how the procedure affected pain levels and functional recovery, a paired *t* test was used to analyze the changes in both the VAS score and the AOFAS score. A significance level of .05 (α) was chosen for all statistical tests. The analysis was conducted using SPSS software, version 26.0 (IBM, Armonk, NY). The institutional review board reviewed the study protocol and ensured that it met ethical standards, including informed consent, participant safety, and data privacy, according to the National Institutes of Health.

Initially, all patients underwent conservative management of OCLTs, including rest, casting immobilization, physical therapy, and nonsteroidal anti-inflammatory drugs for pain.^9^^,^^30^ Patients who did not respond to this conservative approach were offered surgical intervention.

**Fig 4:**
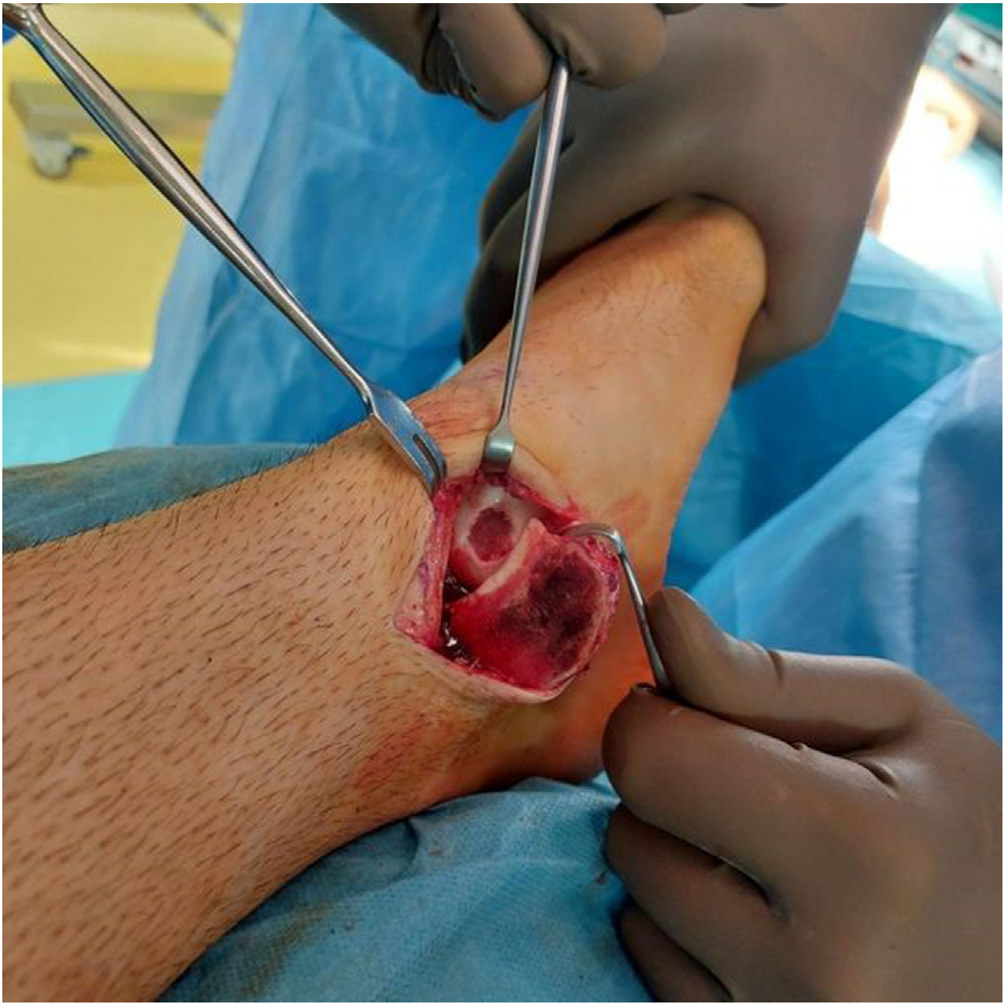
Intraoperative photograph showing preparation of the recipient site on the medial side of the talus for autograft placement.

A diagnostic arthroscopy was performed before the surgical procedure to locate any impingement or loose bodies. Chondroplasty was performed arthroscopically through anteromedial and anterolateral portals to remove any free edematous debris or impinging tissue.^31^ Loose chondral or osteochondral fragments were removed, and a mechanical shaver was used to smooth the damaged cartilage and create a stable articular surface.^31^ For medial lesions for which a medial approach was required, 2 small screw holes were predrilled to aid in malleolar reduction after the procedure.^17^ OATS was then performed using an open approach under spinal anesthesia. Osteotomy was planned based on the location of the lesions. For medial lesions (anatomic locations 4 and 7 on the Raikin grid), a longitudinal incision was made to the projection of the medial malleolus and extended posteriorly and distally to expose the underlying surface. A medial malleolar osteotomy was then performed^16^^,^^21^^,^^32^ (Fig 4). For lateral defects, a longitudinal incision was performed directly over the lateral malleolus. Because of the anterolateral location of the lesion, access was obtained without the need for a tibial osteotomy; only a fibular osteotomy was required, and the tibia was distracted.^16^^,^^21^^,^^32^ In a plantar-flexed position, the anterolateral capsule was resected, and the joint was subluxated to expose the lesion.^16^^,^^21^^,^^32^ The lesions were prepared with debridement of the affected area along with the removal of any loose cartilage.

**Fig 5:**
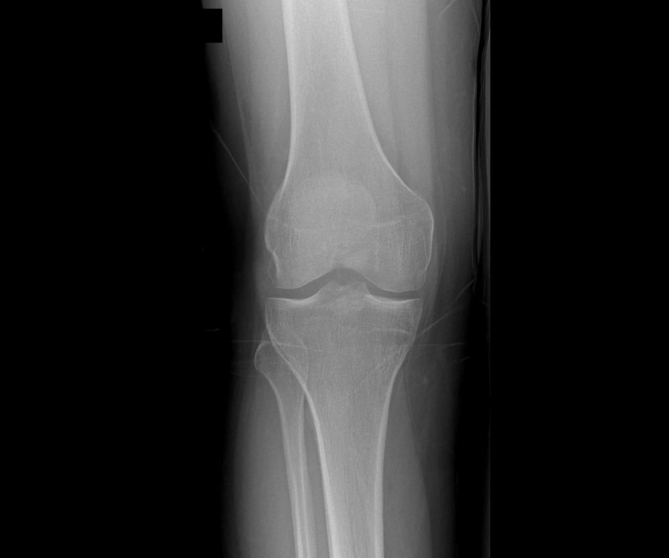
The integrity of the joint and donor site is verified on a preoperative radiograph of the knee.

The autograft OATS 2.0 Set (Arthrex, Naples, FL) was used to estimate the size and number of grafts required for transplantation.^16^ A preoperative radiograph of the knee was obtained to verify the integrity of the joint and donor site (Fig 5). Grafts were obtained through an open surgical technique from the periphery of the ipsilateral lateral femoral condyle of the knee.^24^ An incision was made along the superolateral border of the patella, and a small arthrotomy was performed. Two surgical retractors were positioned to expose the sulcus terminalis and to harvest the graft.^33^ A set of sizers with diameters of 4.5, 6, 8, and 10 mm were used to determine the defect size precisely.^16^ A donor tube harvester was assembled to match the sizers, and it was gently driven into the subchondral cartilage of the ipsilateral lateral femoral condyle to a depth of 15 mm using a mallet.^33^ After reaching the appropriate depth, the harvester was rotated before being driven out with the donor cartilage inside.

The defect was over-drilled with an acorn-shaped drill tip to achieve a depth on average of up to 15 mm.^34^ An alignment stick with an appropriate diameter was used to measure the depth and alignment of the recipient socket. The donor tube harvester was guided into the recipient socket, and the core extruder was gradually turned clockwise into the tube, pushing the donor cartilage inside the recipient socket.

**Fig 6:**
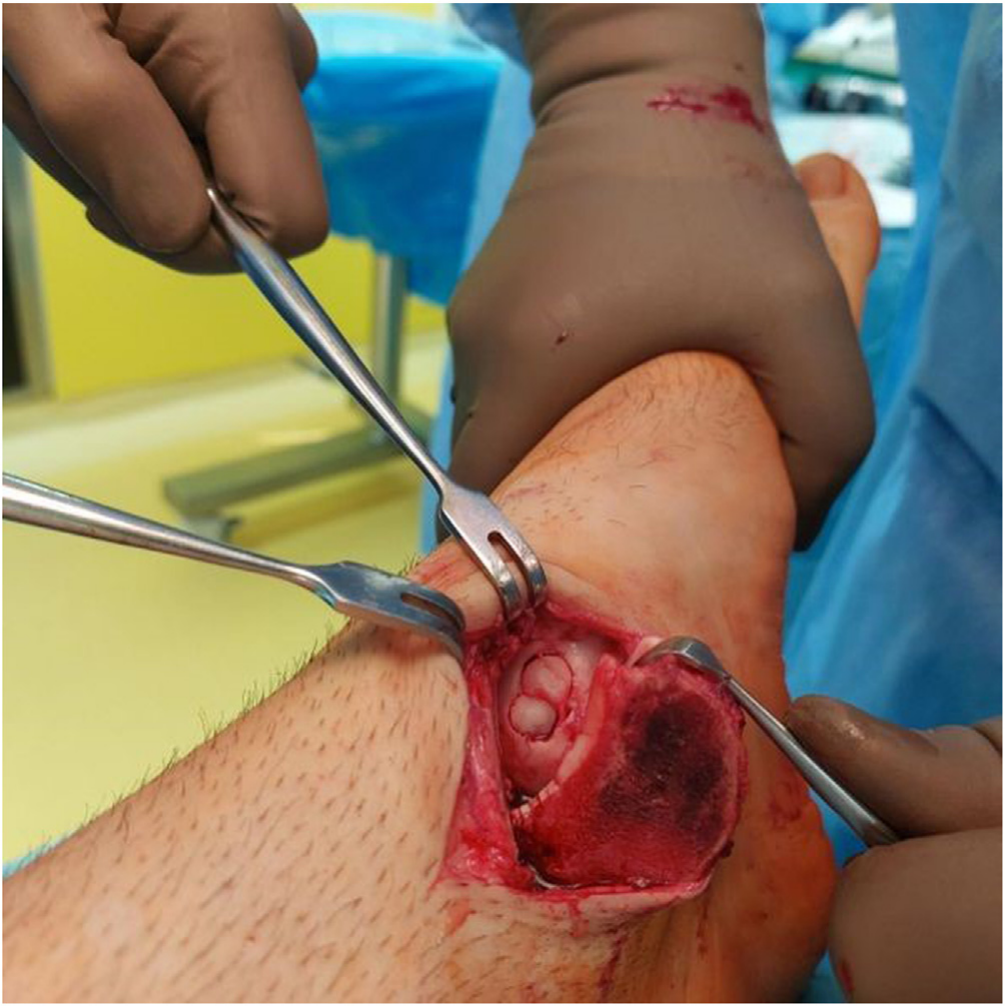
Intraoperative photograph showing insertion of the autograft after preparation.

The graft was carefully impacted by gentle tapping using a mallet until it was flush with the surrounding surface or a maximum of 1 mm deeper (Fig 6). For defects more significant than 10 mm, additional grafts were individually placed, one at a time, ensuring that all steps were followed, from harvesting to placing the graft in the recipient socket.^16^ In 21% of cases, small gaps were present between the grafts during OATS and additional chondroplasty was performed; these remaining defect spaces were filled with a combination of PRP and the remnants of cartilage collected during the procedure. A matrix was not used to stabilize the PRP; instead, during the OATS procedure, a Spongostan sponge (Johnson & Johnson, New Brunswick, NJ) was used to promote fibrin deposition and serve as a biological scaffold, thereby enhancing defect filling and stabilizing PRP.^35^ The PRP harvesting and purification technique was based on the technique proposed by Cugat et al.^36^ The PRP isolates we used had a high platelet concentration, with a low leukocyte count and a low erythrocyte count.^37^

**Fig 7:**
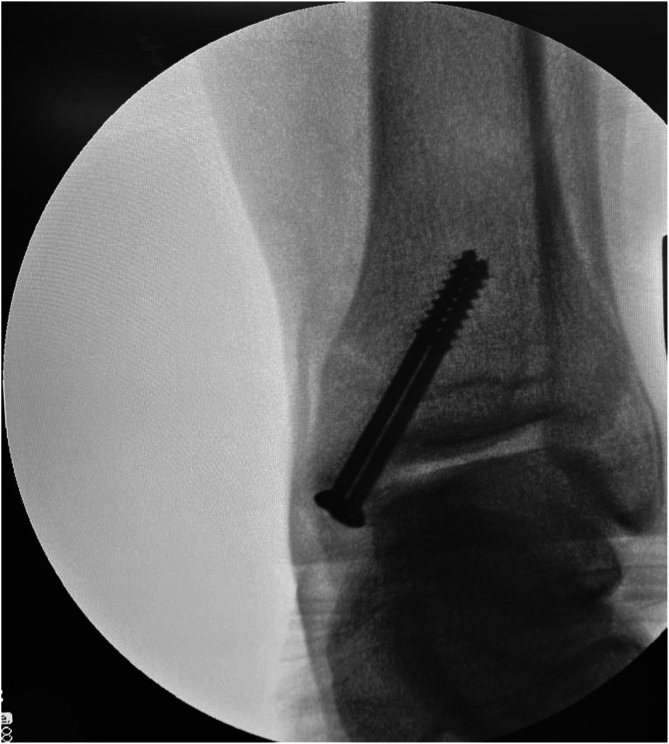
Intraoperative fluoroscopic image confirming the correct position of the osteotomy.

Once the defect was flush with the surrounding cartilage, the tibiotalar joint was reset, and the medial malleolus was fixated and reattached using two 3.5-mm cortical or lag screws. The pre-existing screw holes were used to secure, reattach and reduce the medial malleolus.^16^ The lateral malleolus was reduced and fixed with a 3.5-mm tubular plate. Finally, fluoroscopy was performed intraoperatively to verify the correct positioning of the osteotomy^16^ (Fig 7).

Postoperative management of the tibiotalar joint consisted of immobilization using a posterior splint with strict elevation for 2 weeks. This was followed by 4 to 6 weeks of non-weight-bearing using crutches and a controlled-ankle-movement boot, with partial weight-bearing exercises confined to the boot for an additional 2 to 3 weeks.^34^ During the first 6 weeks, ankle exercises with limited range of motion were encouraged, progressing to full active range of motion and full weight bearing thereafter.^34^

All patients received 3 intra-articular PRP injections into the tibiotalar joint, administered once every 2 weeks for 6 weeks in total, followed by HA injections every 6 months for 2 years.^30^ The patients’ follow-up schedule to evaluate their functional status did not coincide with their scheduled visits to receive PRP and HA injections.

Routine clinical examinations were performed the day after surgery, and wound assessment was performed at 10 to 12 days postoperatively.^34^ Postoperative follow-ups were performed at 4 to 6 months, 12 months, and 24 months.^13^ During each follow-up appointment, patients were evaluated for complications such as pain and stiffness, as well as range of motion of both the ankle and knee joints, and their responses were recorded.^13^ Patients self-reported restriction of motion using questions from the AOFAS questionnaire; we confirmed whether any restriction of motion was present using a universal goniometer and inclinometer.^38^ Postoperative radiographs of the ankle joint were obtained during the 4- to 6-month follow-up.^13^ Clinical assessment involved the administration of the VAS and AOFAS questionnaires to measure pain and range of motion, respectively, during preoperative evaluation and again at 12 months postoperatively.^16^^,^^32^^,^^39^

**Fig 8:**
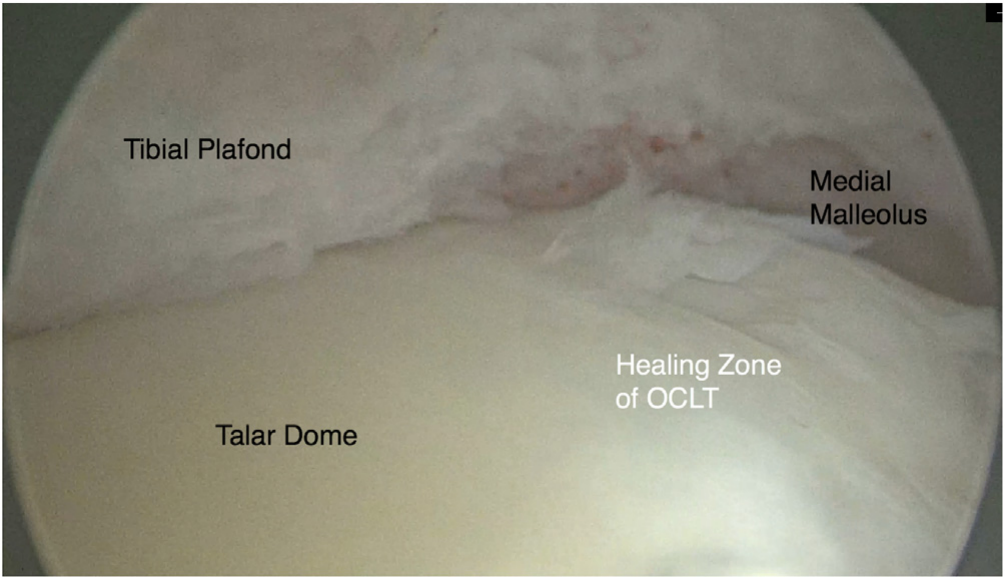
Postoperative arthroscopic evaluation to examine graft site healing. A view of the tibial plafond and the cartilaginous surface of the talar dome from the anterolateral portal shows an intact surface, with healing of the lesion. (OCLT, osteochondral lesion of talus.)

At the 12-month follow-up, second-look arthroscopy and hardware removal were performed as per our institutional policy and the personal preference of the patient, as well as the surgeon. On second-look arthroscopy, healing of the tibiotalar joint was assessed and graft integration was examined^11^ (Fig 8). With the patient under general anesthesia, the ankle was fully dorsiflexed. An anteromedial skin incision was made, and a standard 4-mm, 30° arthroscope was used. Distraction was avoided because of potential tightening of the anterior capsule and hindered visualization. An anterolateral portal was made under arthroscopic guidance, and additional portals (anterolateral or anteromedial near the malleolus or posterolateral) were used as required.^40^ If adhesive scar tissue was identified, debridement of the intra-articular fibrotic tissue was performed to promote more effective rehabilitation and outcomes in the future. In all patients who underwent OATS with chondroplasty, the screws were removed to ensure that hardware would not hinder future imaging and to enable MRI evaluation of potential complications.

## Results

This study included 19 patients (12 male and 7 female patients) who were treated for OCLTs. All 19 patients underwent OATS with or without chondroplasty (14 patients underwent osteotomy with chondroplasty plus OATS, 4 patients underwent osteotomy alone plus OATS, and 1 patient with an anterolateral lesion underwent chondroplasty plus OATS without osteotomy); 3 patients who underwent chondroplasty alone were excluded from this study. The mean age was 31 years (range, 20-53 years), and the mean body mass index was 27 ± 4. Eighteen patients had medial OCLTs (corresponding to 4 and 7 on the Raikin grid), whereas only 1 patient had a lateral OCLT (corresponding to 3 and 6 on the Raikin grid). All patients received the complete PRP and HA protocol, and the mean total follow-up period was 3 years.

All patients showed statistically significant improvements in the VAS score and AOFAS score at the 12-month follow-up (Table 2). At the 24-month follow-up, all patients self-reported a full range of motion and no pain, as well as a complete return to previous preoperative activity. VAS and AOFAS scores were not obtained at 24 months; we only asked an anchor question, on the basis of which we conducted an analysis of substantial clinical benefit (SCB) and patient acceptable symptom state (PASS). The VAS score increased from an average of 56.46 preoperatively to 89.87 postoperatively. The mean increase was 33.40 (95% confidence interval, 30.9-35.8). Similarly, the AOFAS score significantly improved, with the mean score increasing from 52.49 preoperatively to 89.99 after surgery. A mean improvement of 37.49 (95% confidence interval, 34.7-40) was recorded for the AOFAS score. Male patients, on average, had improved VAS scores when compared with female patients, with a mean difference of 3.7 ± 3.9 (*P* < .05); however, the mean change in AOFAS scores, with an improvement of 0.4 ± 3.7, was not statistically significant (*P* > .05).

**Table 2:** Comparison of Mean Preoperative and Postoperative VAS and AOFAS Scores

	VAS Score	AOFAS Score
Mean score		
Preoperative	56.46	52.49
Postoperative	89.87	89.99
Change in score, mean ± SD	33.40 ± 5.44	37.49 ± 6.00
*P* value	<.001	<.001

NOTE. *P* < .05 was considered significant.

AOFAS, American Orthopaedic Foot & Ankle Society; SD, standard deviation; VAS, visual analog scale.

In a cohort-specific analysis of the minimal clinically important difference, the threshold values were 3 and 2.72 for the VAS score and AOFAS score, respectively; 100% of our patients met or exceeded the threshold values for the VAS and AOFAS scores. We conducted an analysis of the PASS and SCB based on the following anchor question at the 24-month follow-up: “Did you return to your pre-operative activity level?” According to our question-based PASS and SCB analysis, 100% of patients reported returning to their preoperative activity levels. We also conducted an SCB analysis based on thresholds established in the literature (≥30-point improvement in VAS score and ≥34-point improvement in AOFAS score); 74% of patients met or exceeded the threshold for VAS score improvement, and 72% of patients met or exceeded the threshold for AOFAS score improvement.^41^

**Fig 9:**
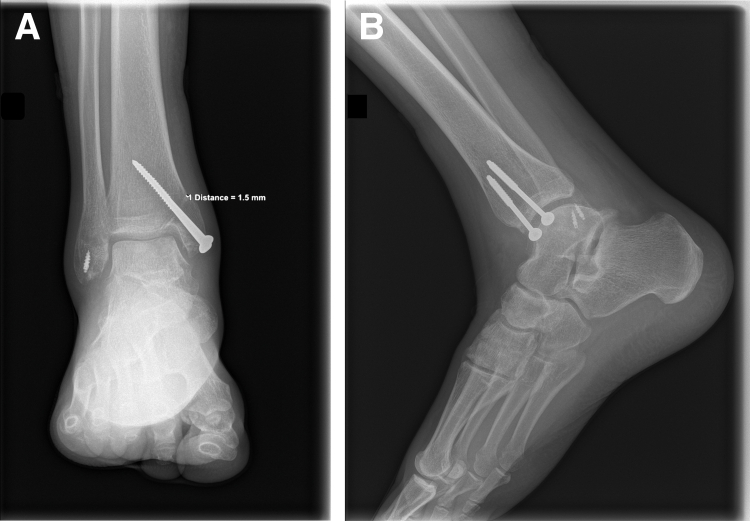
Postoperative anteroposterior (A) and lateral (B) views showing screw placement.

No postsurgical complications or cases of delayed healing or nonunion were reported in patients who underwent malleolar osteotomy. A total of 9 patients (45%) complained of knee pain at the 4- to 6-month follow-up, which eventually subsided without any intervention after 12 months. Ankle radiographs obtained at the 4- to 6-month follow-up revealed complete union of the malleolar osteotomy and no observable osteochondral abnormalities (Fig 9). Of 19 OATS patients, 17 reported no restriction of motion at the 12-month follow-up; however, 2 of 19 patients reported mild restriction of motion with dorsiflexion at the ankle joint. Second-look arthroscopy showed complete healing of OCLTs with full incorporation of the graft (Fig 8). At the 24-month follow-up, all patients reported a full range of motion and no pain, as well as a complete return to previous preoperative activity.

## Discussion

In this study, all patients with OCLTs returned to preinjury activity levels after treatment with OATS combined with PRP and HA injections. On the basis of radiologic findings, OCLTs are staged and graded according to the Berndt and Harty classification system and/or Hepple classification system to guide management.^2^^,^^13^ Alternatively, the Pritsch classification system uses arthroscopy to evaluate and stage OCLTs.^3^^,^^18^

The VAS score and AOFAS score are frequently used assessment tools for evaluating the severity and outcomes of OCLTs.^3^^,^^34^^,^^42^ We used the VAS score because it is a relatively straightforward, patient-centered tool and has been well supported by the literature.^34^^,^^42^ The AOFAS score is another comprehensive scoring system specifically used in patients with hindfoot and ankle conditions that aims to evaluate pain, mobility, and foot alignment.^42^^,^^41^ Both scores are well supported by the literature for tracking patient progress, evaluating treatment efficacy, and facilitating outcome comparisons.^3^^,^^18^^,^^41^^,^^42^

The Raikin anatomic grid is a commonly applied and well-validated anatomic scheme used to localize OCLTs with respect to their anterior-posterior and medial-lateral position, as seen on MRI.^4^^,^^43^ OCLTs are more commonly located medially in locations 4 and 7 on the Raikin grid, corresponding to our findings.^4^^,^^43^ However, it is notable that most of our patients (91%) had medial OCLTs compared with the 58% to 63% figure that is most commonly reported.^4^^,^^44^^,^^45^

The effectiveness of PRP in treating OCLTs has been validated extensively in the literature.^21^^,^^46^ Mei-Dan et al. conducted a randomized controlled trial showing improved outcomes measured by the VAS score with PRP injections in patients treated nonoperatively.^47^ Additionally, a systematic review by Woo et al.^21^ indicated superior outcomes when combining PRP with microfracture compared with either treatment alone. Similarly, Guney et al.^20^ reported significantly improved pain and functional outcomes in patients treated with microfracture plus PRP versus microfracture alone. A study published by Görmeli et al.^22^ investigating the outcomes of PRP versus HA versus control after microfracture surgery for the treatment of OCLTs showed that patients treated with PRP or HA after microfracture surgery had improved postoperative pain and functional outcomes compared with control patients. They observed greater improvement in the PRP group than in the HA group.^22^

HA has also been validated as effective in enhancing postoperative outcomes for OCLTs, as evidenced by notable studies conducted by Doral et al.^48^ and Shang et al.^49^ The randomized controlled trial by Görmeli et al.^22^ further supported these findings, highlighting improved postoperative pain and function with PRP and HA treatments after microfracture. Although there is currently limited literature specifically addressing the combined use of PRP and HA in osteochondral autologous transplantation for OCLTs, this combination has shown success in patients with knee osteoarthritis, as observed in a systematic review by Karasavvidis et al and a narrative review by Gilat et al.^50^, ^51^ In our case series, we combined PRP and HA adjunctively, achieving favorable outcomes, and these promising outcomes suggest that there is potential value in conducting a comprehensive randomized controlled trial to test these prelimnary results on a larger scale. Further developing procedure-specific guidelines that integrate PRP and HA therapies may further assist surgeons in optimizing outcomes for patients with OCLTs.

The goal of OATS is to repair defects by introducing a graft that resembles the mechanical, structural, and biological properties of the patient’s native hyaline cartilage.^4^ OATS has shown promising results for medial and lateral talar lesions; however, limitations have been observed for OCLTs located at the central talus.^30^ A systematic review by Shimozono et al.^52^ reported improvements in AOFAS ankle and hindfoot scores from 55.1 preoperatively to 86.2 postoperatively, with 87% of patients showing good to excellent results.^29^^,^^30^ Several retrospective case series have reported similar clinical outcomes with significant improvement in postoperative short- and long-term VAS scores.^53^^,^^54^ Complications associated with OATS include donor-site morbidity, symptomatic hardware, infection, anterior ankle impingement, nerve injury, and nonunion of the graft.^52^ Other potential complications are related to the associated osteotomy, including delayed union, nonunion of the malleolus, or progression to arthritis.^16^ The most common complication is donor-site morbidity, with a general incidence of 10.6% according to a systematic review published by Shimozono et al.^46^ A meta-analysis with best-case and worst-case analysis conducted on 26 studies estimated the proportion of donor-site morbidity to range from 6.7% to 10.8%.^30^^,^^46^

The existing literature on optimal donor sites for harvesting grafts is limited; however, the most commonly used graft sites are the intercondylar notch and the lateral femoral condyle.^15^^,^^16^^,^^30^ Other less commonly used donor sites include non-weight-bearing regions of the talar joint and proximal tibiofibular joint.^14^ The grafts for our study were obtained from the lateral femoral condyle of the ipsilateral knee because the surface variation resembles that of the talar dome; in addition, up to 3 grafts can be harvested without compromising the patellofemoral joint.^16^ The most frequent complaint reported by patients was knee pain at the donor site, which was observed in 47.3% of the patients during the 4- to 6-month follow-up period. Other symptoms that increase the risk of donor-site morbidity include instability, discomfort, crepitus, effusion, and stiffness; however, these were not reported by the patients in our study.^30^^,^^46^, ^55^

Shimozono et al. reported the requirement for additional procedures, including arthroscopy with debridement, ankle fusion, symptomatic hardware removal, and revision OATS, in up to 6.2% of patients.^52^ Clinical failure requiring ankle fusion and revision surgery was reported in only 1% of patients.^46^^,^^52^ Similar findings have been reported by Winkler et al.^56^ Their retrospective study conducted in 2023 evaluated long-term clinical and radiologic outcomes of patients undergoing OATS and reported that 17.1% of patients met the criteria for clinical failure at 12.2 ± 6.6 years after OATS.^56^ Winkler et al. also conducted a survival analysis and showed an estimated mean survival time of 21.3 years and a 20-year survival rate of 77.9%. On the basis of our findings, we believe that the minimal complication rate was because of routine removal of hardware and second-look arthroscopy, which allowed for more aggressive rehabilitation and thus decreased the risk of these potential complications.^57^, ^58^, ^59^

### Limitations

Our study is not without limitations, including the relatively small sample size of patients, which may have affected data synthesis, as well as the retrospective nature of the study. Another important limitation is that this study lacked a control group for comparison, which may have limited the interpretation of our results. Additionally, the lack of access to biosynthetic graft material in a developing country may have contributed to an increased incidence of donor-site morbidity.

## Conclusions

Combining OATS with PRP-HA injections can yield promising results for patients with OCLTs, showing significant improvement in VAS and AOFAS scores postoperatively.

## Disclosures

All authors (G.A.R., N.P., M.A.M., T.T., G.K., I.G., I.K., G.Z., G.L., V.G., M.Z., L.N.) declare that they have no known competing financial interests or personal relationships that could have appeared to influence the work reported in this paper.
